# Dataset on the effect of different pretreatment on the proximate analysis, microbial and sensory evaluation of dried banana during its storage

**DOI:** 10.1016/j.dib.2020.105678

**Published:** 2020-05-13

**Authors:** Edith E. Alagbe, Ebakota O. Daniel, Esther A. Oyeniyi

**Affiliations:** a Department of Chemical Engineering, Covenant University, Ota. Nigeria; b Department of Microbiology, Benson Idahosa University, Benin City. Nigeria

**Keywords:** microbial loads, oxidative reactions, post-harvest loss, proximate analysis, shelf life studies, water activity of food

## Abstract

Pre-treatment on banana slices is usually carried out to stop discolouration of the fruit by oxidative reactions, in raw presentation of the fruit especially in fruit salads. Drying is an old long preservation method for fruits and vegetables. In drying banana fruit, discolorations do occur and an attempt to stop the discoloration while maintaining quality (shelf life) is studied in this work. Dataset presented here, is on the effect of different pretreatment on the proximate analysis, microbial and sensory evaluation of dried banana during its storage. The pre-treatment methods considered in this work, were lemon juice and carbonated lemon drink while the control had no pre-treatment on the slices before drying. Proximate analysis and water activity of raw and dried samples were carried out while the microbial and sensory evaluation changes were observed in the samples over one month period.

Specifications table

**Table:** 

Subject	Agricultural and Biological Science
Specific subject area	Food Science: Shelf life studies
Type of data	Tables
How data were acquired	The modified method of Alagbe et al. [1] was followed for the pretreatment and subsequent drying of the banana slice. 30g of dried banana were packed in 12 cm x 8.85 cm resealable plastic packs and refrigerated at a temperature and relative humidity of 4 ± 2 ^0^C and 55% respectively. Analyses of samples were carried out on the packed samples every week and for four weeks (one month). Parameters monitored were the water activity as evaluated from the works of Alagbe et al. (unpublished) using existing models [2,^3^,4], ash content, percent crude protein and fibre content, were determined according to method described by A.O.A.C [5]; the carbohydrate content was determined using the difference method; the pH of the samples were determined using a pH meter (Hanna microprocessor). Microbial presence was also evaluated on the samples using standard methods.
Data format	Raw
Parameters for data collection	Drying of the banana slices was carried out at 65 ^0^C, for 18 hours, cooled and packed in sealed plastic packs.
Description of data collection	Determination of water activity, ash content, fibre and carbohydrate content and microbial presence.
Data source location	Ogun State, Nigeria.
Data accessibility	Data is available with the article

## Value of the Data

• These data are useful for insight into the effect of pre-treatment in the shelf life of dried banana samples.
• Food scientist, nutritionist and equipment fabricators can benefit from this data. Also, this work is beneficial to the Small, Medium Enterprises.
• The data can provide useful information on the level of preservation needed for the dried slices for any specific period of time.

## Data Description

The shared data is about the effect of different pretreatment on the proximate analysis, microbial and sensory evaluation changes of tray dried banana slices during its storage. Drying of the banana slices was effected in a tray dryer at a temperature of 65^0^C for 18 hours. The results of the proximate analysis, rate at which moisture is reabsorbed by the dried banana slices, changes in pH during storage and changes in the Titrable Acids (TTA) during storage are presented in Tables 1 – 4 respectively.

## Experimental Design, Materials, and Methods

### Sample preparation

Raw banana fruits were peeled and sliced into cylindrical shapes of about 5 mm thickness, using a banana slicer according to the modified method of Alagbe et al. [1]. The Eureka lemon (*citrus limon ‘Eureka’)* was used in this work and the lemon juice was prepared in a 1:4 ratio (being volume of lemon extract to volume of water) and samples labeled, L while the carbonated lemon drink was purchased Sprite brand with samples labeled, S. The non-treated (raw) banana (designated, C) served as the control.

200g each of sliced banana were pre-treated by dunking for 10 minutes in 400ml of lemon juice (designated, L) and carbonated lemon drink (designated, S) and after 10 minutes, the treated slices were sieved and mopped with filter paper to remove the excess water on the surface of the slices.

**Table 1:** Results of proximate analysis of raw banana and of the dried banana samples after one month.

	Fresh sample	Sample (C)	Sample (L)	Sample (S)
Ash (%)	2.86	1.94	1.87	1.46
Carbohydrate (%)	8.59	1.86	1.39	1.27
Protein (%)	0.09	2.74	1.30	1.68
Fibre (%)	12.60	70.27	69.15	68.65
Lipids (%)	0.08	19.67	22.31	23.49

**Table 2:** Variation of water activity in dried banana, with time during storage.

WEEK	Dried Banana Samples / Water activity, %		
	C	L	S
0	0.19	0.19	0.21
1	0.30	0.32	0.49
2	0.69	0.62	0.61
3	0.82	0.84	0.85
4	0.95	0.96	0.93

**Table 3:** Changes in pH of dried samples over time during storage.

WEEK	Dried Banana Samples / pH		
	C	L	S
0	3.35	3.42	3.58
1	3.36	3.54	3.61
2	4.67	4.24	4.14
3	5.62	5.49	5.42
4	5.68	5.50	5.52

**Table 4:** Changes in the total Titrable Acids (TTA) in the dried samples during storage.

WEEK	Dried Banana Samples / TTA (%)		
	C	L	S
0	30.48	71.02	55.36
1	29.76	69.44	54.40
2	20.80	50.56	38.59
3	18.20	40.00	36.32
4	17.50	35.12	32.22

**Table 5:** Changes in sensory evaluation properties of the packaged samples after one month.

Properties/Sample	C		L		S	
	Day 1	Day 30	Day 1	Day 30	Day 1	Day 30
Colour	Cream yellow	Tan	Cream yellow	Cream yellow	Cream yellow	Cream yellow
Taste	Sweet	Sweet	Sweet	Sweet	Sweet	Sweet
Texture	Crispy	Soft	Crispy	Soft	Crispy	Soft

**Table 6:** Changes in microbial count of stored and packaged dried banana samples (C) over time.

Days	Count (cfu/g)							
	Hetrotrophic count		*Salmonella* Count	*Shigella* Count	*Escherichia* count	*Staphylococcus* count	Coliform count	Fungi count
0	10 × 10^1^	NIL	NIL	NIL	NIL	NIL	NIL	NIL
3	18 × 10^1^	3 × 10^2^	NIL	NIL	NIL	NIL	NIL	NIL
7	35 × 10^1^	10 × 10^2^	NIL	NIL	NIL	NIL	NIL	NIL
10	58 × 10^1^	21 × 10^2^	NIL	NIL	NIL	NIL	NIL	NIL
14	64 × 10^1^	13 × 10^2^	NIL	NIL	NIL	NIL	NIL	NIL
17	80 × 10^1^	25 × 10^2^	NIL	NIL	NIL	NIL	NIL	NIL
21	110 × 10^1^	52 × 10^2^	NIL	NIL	NIL	NIL	NIL	NIL
24	130 × 10^1^	50 × 10^2^	NIL	NIL	NIL	NIL	10 × 10^1^	9 × 10^1^
28	160 × 10^1^	54 × 10^2^	NIL	NIL	NIL	NIL	15 × 10^1^	20 × 10^1^
31	166 × 10^1^	59 × 10^2^	NIL	NIL	NIL	NIL	25 × 10^1^	25 × 10^1^

**Table 7:** Changes in microbial count of stored and packaged dried banana samples (L) over time.

Days	Count (cfu/g)							
	Hetrotrophic count		*Salmonella* Count	*Shigella* Count	*Escherichia* count	*Staphylococcus* count	Coliform count	Fungi count
0	15 × 10^1^	4 × 10^2^	NIL	NIL	NIL	NIL	NIL	NIL
3	25 × 10^1^	6 × 10^2^	NIL	NIL	NIL	NIL	NIL	NIL
7	30 × 10^1^	10 × 10^2^	NIL	NIL	NIL	NIL	NIL	NIL
10	50 × 10^1^	10 × 10^2^	NIL	NIL	NIL	NIL	NIL	NIL
14	55 × 10^1^	15 × 10^2^	NIL	NIL	NIL	NIL	NIL	NIL
17	80 × 10^1^	33 × 10^2^	NIL	NIL	NIL	NIL	NIL	NIL
21	100 × 10^1^	48 × 10^2^	NIL	NIL	NIL	NIL	NIL	NIL
24	150 × 10^1^	51 × 10^2^	NIL	NIL	NIL	NIL	NIL	NIL
28	187 × 10^1^	55 × 10^2^	NIL	NIL	NIL	NIL	NIL	NIL
31	205 × 10^1^	66 × 10^2^	NIL	NIL	NIL	5 × 10^1^	46 × 10^1^	5 × 10^1^

**Table 8:** Changes in microbial count of stored dried banana samples (S) over time.

Days	Count (cfu/g)							
	Hetrotrophic count		*Salmonella* Count	*Shigella* Count	*Escherichia* count	*Staphylococcus* count	Coliform count	Fungi count
0	5 × 10^1^	NIL	NIL	NIL	NIL	NIL	NIL	NIL
3	10 × 10^1^	2 × 10^2^	NIL	NIL	NIL	NIL	NIL	NIL
7	10 × 10^1^	3 × 10^2^	NIL	NIL	NIL	NIL	NIL	NIL
10	18 × 10^1^	5 × 10^2^	NIL	NIL	NIL	NIL	NIL	NIL
14	28 × 10^1^	9 × 10^2^	NIL	NIL	NIL	NIL	NIL	NIL
17	30 × 10^1^	8 × 10^2^	NIL	NIL	NIL	NIL	NIL	NIL
21	50 × 10^1^	15 × 10^2^	NIL	NIL	NIL	NIL	NIL	NIL
24	75 × 10^1^	33 × 10^2^	NIL	NIL	NIL	NIL	NIL	NIL
28	100 × 10^1^	41 × 10^2^	NIL	NIL	NIL	NIL	7 × 10^1^	15 × 10^1^
31	131 × 10^1^	56 × 10^2^	NIL	NIL	NIL	5 × 10^1^	40 × 10^1^	25 × 10^1^

Drying of samples was accomplished by loading batches of the treated and untreated banana slices into an oven at a temperature of 65 ^0^C, for 18 hours according to works of Alagbe et al. (unpublished).

## Packaging of dried samples

After drying, 50g of each sample were packed in 12 cm x 8.85 cm resealable plastic packs and refrigerated at a temperature and relative humidity of 4 ± 2 ^0^C and 55% respectively. Common handling procedures of the dried banana slices were mimicked for analysis (packs were brought out of the packs and analyzed at room temperature before the remainder were returned to the refrigerator). 2 – 5 g of samples were taken out of the pack every week for analysis according to standard methods [5,^6^,^7^,8].

## Analysis of samples

Water activity, ash content, crude fibre, protein content, total titrable acids (TTA) and sensory evaluation changes of the fresh and dried samples were determined every week and data obtained are presented in Tables 1 – 5 below. The total duration of the packed dried banana slices was 4 weeks.

## Appendix Supplementary materials

Image, application 1
